# Modified Wood Fibers Spontaneously Harvest Electricity from Moisture

**DOI:** 10.3390/polym16020260

**Published:** 2024-01-17

**Authors:** Tao Zhang, Xuewen Han, Yukang Peng, Han Yu, Junwen Pu

**Affiliations:** Beijing Key Laboratory of Lignocellulosic Chemistry, Beijing Forestry University, Beijing 100083, China; ztofcl@163.com (T.Z.); hxw18842664740@bjfu.edu.cn (X.H.); pyukang@163.com (Y.P.); yuhan5339@163.com (H.Y.)

**Keywords:** moisture-enabled, electricity generator, wood modification, ambient air, ionic transport

## Abstract

With the rapid development of modern society, our demand for energy is increasing. And the extensive use of fossil energy has triggered a series of problems such as an energy crisis and environmental pollution. A moisture-enabled electric generator (MEG) is a new type of energy conversion method, which can directly convert the ubiquitous moisture in the air into electrical energy equipment. It has attracted great interest for its renewable and environmentally friendly qualities. At present, most MEGs still have low power density, strong dependence on high humidity, and high cost. Herein, we report the development of a high-efficiency MEG based on a lignocellulosic fiber frame with high-power-density, all-weather, and low-cost characteristics using a simple strategy that optimizes the charge transport channel and ion concentration difference. The MEG devices we manufactured can generate the open-circuit voltage of 0.73 V and the short-circuit current of 360 μA, and the voltage can still reach 0.6 V at less than 30% humidity. It is possible to drive commercial electronic devices such as light-emitting diodes, electronic displays, and electronic calculators by simply connecting several electric generators in series. Biomass-based moisture-enabled electric generation has a low cost, is easy to integrate on a large scale, and is green and pollution-free, providing clean energy for low-humidity or high-electricity-cost areas.

## 1. Introduction

Against the backdrop of increasing global demand for clean energy, research on developing renewable energy and efficient energy conversion technologies has become increasingly important [1,2]. Traditional power generation methods often rely on fossil fuels or nuclear energy, which poses many environmental and safety issues [3,4,5]. In order to obtain clean energy, researchers have made tremendous efforts to develop various energy conversion systems based on principles such as piezoelectric, triboelectric, and thermoelectric principles [6,7,8]. Moisture-enabled electric generation (MEG) is different from the traditional hydroelectric and hydropower generation methods [9,10]. The device can spontaneously absorb moisture in the air, interacting with the oxygen-containing functional groups in the active material to release charged ions and migrate with the moisture in the moving material to generate electrical energy. The structure is to sandwich the active material with oxygen-containing functional groups in the middle of an asymmetric electrode, forming a sandwich structure. This emerging method of green energy harvesting, by converting ubiquitous airborne moisture into electricity, is attracting the attention of researchers for its sustainability, simple device construction, ease of preparation, and portability [11,12]. In recent years, researchers have developed various materials for making MEGs, such as carbon-based materials, metal oxides, polymers, bio-fibers [13,14,15,16]. The principle of designing MEG is roughly to utilize mechanisms such as a redox reaction, ion gradient diffusion, and a streaming current to enable functional groups in the material to interact with water, dissociate protons, and generate electricity through the directional migration of protons [12,17]. However, currently, most designed MEGs have issues such as limited output power, inability to continuously output, and the need for high-humidity environments that urgently need to be addressed. Gao et al. [18] constructed a paper-based generator using printed paper as a wet–electric material to produce ions through dissociation of paper oxygen-containing functional groups to obtain an open-circuit voltage of 0.25 V. However, the intermittent voltages produced by this device could not meet the application requirements. Xu et al. [19] used asymmetric deposition of an ionic hydrogel on the surface of graphene in order to obtain MEGs and derive electrical power from atmospheric moisture, but the generator needed to be operated under 98% of RH extreme humidity to obtain an open-circuit voltage of 0.34 V and a short-circuit current of a 1 μA output power of only 0.33 μW. Therefore, obtaining a stable and continuous output with high power under daily humidity is the current research hotspot for moisture generators.

Wood is one of the most widely distributed renewable resources in nature [20,21,22]. It fully meets the demand of society for low-cost, environmentally friendly, and high-performance composite materials. The natural anisotropic porous structure of wood allows for the transport of water and ions from the roots to the crown, which is essential for improving the ion migration efficiency of MEG. Wood is mainly composed of cellulose, hemicellulose, and lignin [23,24]. The hydroxyl and carboxyl groups in cellulose molecular chains are the main reactive sites in wood-based materials [25,26]. Such kinds of porosity and reactivity make wood a potential material for preparing MEG. Through a simple modification of natural wood after delignification, Cai et al. [27] provided opposite charge ions for carboxylation and quaternization of wood fibers, increasing the potential difference on the wood surface. Therefore, they obtained an asymmetric double-layer structure of moisture-enabled electric generation. At a relative humidity of 85%, the electric generator generated an output voltage of 0.570 V and a 15 μA short-circuit current. However, the device had a high internal resistance, so the short-circuit current was low. The current of most MEGs prepared is only a few dozen microamperes, unable to meet the needs of practical applications. Researchers are also constantly trying to reduce the internal resistance of MEG devices and increase the current by introducing conductive components and adjusting ion transport paths. Some researchers improved ion transfer efficiency and reduced the internal resistance of generators by effectively regulating ion transfer channels. Eun et al. [28] developed an efficient moisture-enabled electric generation by using a laser engraving machine to graphitize CNF and impregnate it with NaCl. This structure allowed for more water channels between electrodes to transport ions, thus increasing the concentration gradient of NaCl ions. The electric generator generated an output voltage of 0.650 V and a circuit current of 550 μA in the environment with a humidity of 90%. During this process, six device components connected in series lit up four LED bulbs. The electric generator prepared using this method significantly increases the short-circuit current, but it requires high environmental humidity to achieve optimal output, making it unable to meet applications in different regional environments (such as arid areas and water scarcity areas).

Poly (3,4-ethylenedioxythiophene)—poly (styrene sulfonic acid) (PEDOT:PSS) is a highly conductive polymer with excellent conductivity [29]. Having lower impedance and higher charge injection capability than metals, it is widely used in various electronic devices. Wang et al. [30] obtained directional evaporation-driven generators with optimized ion-transport channels by directly coating PEDOT:PSS on the surface of wood fibers. Due to their failure to functionalize the fiber surface to enable the generators to produce sufficiently large amounts of ions in the moisture, there was only a voltage of 0.385 V and 11 μA of current. Both high ion concentration differences and low transport resistance are indispensable factors for increasing the output of moisture generators [31,32].

We removed hemicellulose and lignin from natural wood but retained the complete cellulose skeleton. Then, we loaded carbon black nanoparticles onto the fiber skeleton to obtain carbon black wood (CBW). Therefore, we successfully improved the surface electronegativity of the fiber and increased a large number of nanopores. Introducing it into MEG can improve the transmission efficiency of electrons and plays an important part in solving the high internal resistance of MEG [30]. At the same time, the addition of phytic acid (PA) and LiCl can dissociate enough ions in low-humidity environments to ensure that a large ion concentration difference is always maintained within MEG. Then, by immersing carbon black wood in a solution of PEDOT:PSS and PA in an appropriate ratio, we obtained a conductive layer on the surface of carbon black wood. Finally, LiCl was used as a hygroscopic agent to cover the surface of wood pores and capture moisture in the air. In this work, the wood-based composite (PEDOT:PSS/PA-CBW) MEG we obtained has excellent output power and can maintain good output characteristics even at low humidity, overcoming the obvious shortcomings of MEG at present. It not only provides strategies for optimizing the quality of MEG in the past, but also expands the practical application range of MEG.

## 2. Materials and Methods

### 2.1. Materials

Natural Balsa wood (NW) was purchased from Zhejiang Province, China. Sodium chlorite (NaClO_2_), acetic acid (CH_3_COOH), citric acid, hydrochloric acid, sulfuric acid, phytic acid (PA, 50%), PEDOT:PSS (1.1 wt% in H_2_O), and polydimethylsiloxane (PDMS) were purchased from Aladdin Biochemical Technology Co., Ltd. in Shanghai, China. Nano-carbon black (CB) was purchased from Cabot Chemical Co., Ltd. (Tianjin, China). Sodium dodecylbenzene sulfonate (SDBS) and LiCl were from Beijing InnoChem Science & Technology Co., Ltd. (Beijing, China). The copper sheet and copper mesh were purchased from Beijing Hardware Market (Beijing, China). All chemical reagents were analytical.

### 2.2. Sample Preparation

#### 2.2.1. Carbon Black Wood Preparation

Natural wood was cut along the growth direction and divided into several 1 cm× 1 cm × 0.3 cm blocks. The wooden blocks were cleaned using deionized water and air-dried at room temperature. Sodium chlorite was added to deionized water to prepare a 5 wt% sodium chlorite solution. The pH of the solution was adjusted to 4.6 with acetic acid. The wood blocks were heated and dried in this solution at 80 °C for 6 h for delignification treatment. Then, the wood blocks were rinsed with deionized water three times and freeze-dried to obtain delignified wood (DW). Later, 1gCB and 1gSDBS were mixed and put in 100 mL of deionized water. The water was sonicated for 1.5 h to obtain uniformly dispersed and stable carbon black ink. Under vacuum conditions of a 0.1 bar pressure, delignified wood was impregnated in the carbon black solution for 20 min and dried at 60 °C for 6 h, and the above steps were repeated several times to obtain carbon black wood (CBW).

#### 2.2.2. MEG Preparation

First, PEDOT:PSS and PA were mixed into deionized water in a ratio of 30 wt% to form a mixed solution. The sample of carbon black wood was soaked in the mixed solution for 20 min and freeze-dried to obtain composite carbon black wood (PEDOT:PSS/PA-CBW). Then, PEDOT:PSS/PA-CBW was impregnated in a 10 wt% LiCl solution for 2 h and dried at 60 °C for 6 h. Finally, PEDOT:PSS/PA-CBW was put between equally sized copper sheets and copper grids. The open electrode was at the top and the closed electrode was at the bottom. The top electrode and the bottom electrode were connected with two wires to complete our MEG. The MEG device was encapsulated with polydimethylsiloxane (PDMS) around it to ensure sealing performance.

### 2.3. Sample Preparation

The morphology of natural wood, carbon black wood, and composite carbon black wood was analyzed by using field emission scanning electron microscopy (SU8100, Hitachi, Tokyo, Japan). Fourier Transform Infrared Spectroscopy was measured by using an FTIR analyzer (Nicolet iS20, Thermo Fisher Scientific, Waltham, MA, USA), and a functional group analysis was performed on the sample. Each test was conducted with air as the background and a total of 64 scans were performed, with a resolution of 0.09 cm^−1^ and a spectral range of 4000–600 cm^−1^. Then, the crystal structure of wood samples was characterized by using an X-ray Diffraction Instrument (UltimaIV, Rigaku, Japan). During the process, a Cu-Kα radiation X-ray source was used. The scanning range was 10°–80° and the scanning speed was 10° min^−1^. The elemental composition and valence states of samples were characterized by using X-ray Photoelectron Spectroscopy (PerkinElmer, Waltham, MA, USA). The pore size distribution of delignified wood and composite carbon black wood was measured by using a Porosity Analyzer (Micromeritics, Norcross, GA, USA). The particle size and Zeta potential of carbon black nanoparticles were measured by using a nanoparticle size and Zeta potential analyzer (Zetasizer Nano ZS90, Malvern, UK). EIS measurement was carried out using an electrochemical workstation (CHI660D, Chenhua Instrument, Shanghai, China): when measuring voltage, the current was set to 0; when measuring current, the voltage was set to 0. The mechanical properties of the sample were tested using a Universal Testing Machine (5982, Instron, Norwood, MA, USA). The output voltage and current were recorded using a digital multimeter (UT890D+, UNI-T). The humidity changes were recorded using a temperature and humidity meter (DELI Group Co., Ltd., Ningbo, China).

## 3. Results

**Figure 1:**
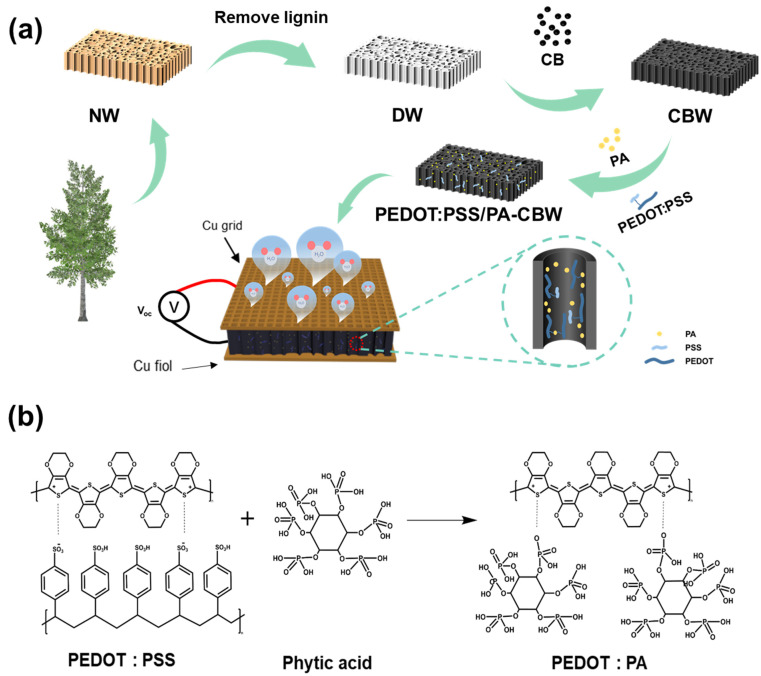
(**a**) Preparation process of the MEG device based on PEDOT:PSS/PA-CBW. (**b**) Reaction formulae for PEDOT and PA.

**Figure 2:**
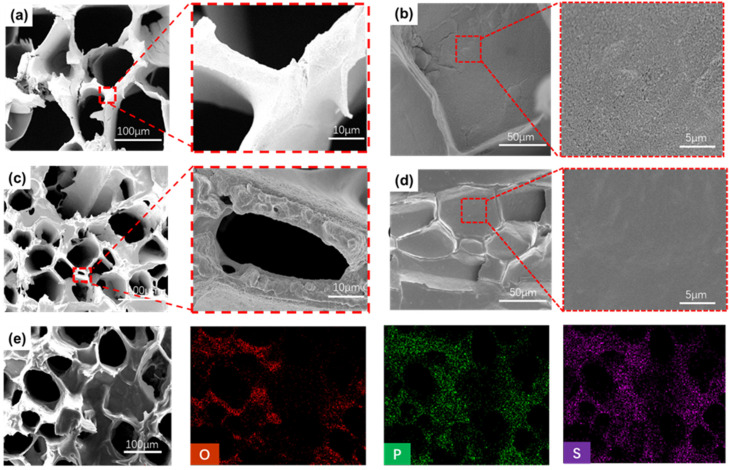
(**a**) SEM images of CBW. (**b**) SEM images of CBW surface. (**c**) SEM images of PEDOT:PSS/PA-CBW. (**d**) SEM images of PEDOT:PSS/PA-CBW surface. FT-IR spectra of DW, CBW, PEDOT:PSS, and PEDOT:PSS/PA-CBW. (**e**) EDS images of PEDOT:PSS/PA-CBW and elemental mapping images of O, P, and S, respectively.

The MEG preparation process and structure we designed are shown in Figure 1. Our design strategy is to utilize the anisotropic channels of wood for transporting water, by modifying them to maximize the number of movable ions and reduce the transmission resistance in the channels. We used the liquid hygroscopic agent LiCl to ensure the supply of water, thereby increasing voltage and current output in the electric generator. Finally, we assembled copper mesh electrodes and copper sheet electrodes into a sandwich structure to ensure that one side of the opening is open to construct a humidity difference (Figure 1a). Firstly, the wood should be treated. Components such as cellulose, hemicellulose, and lignin were contained. The hydrophobicity of lignin was not conducive to water absorption and transportation so it was removed first. The remaining cellulose was the basic framework in this study. As shown in Figure S1, by removing lignin from natural wood, most hydrophobic lignin was removed in order to expose more reactive sites and increase the size of micro and nanochannels. Cellulose wood has large interconnected channels that facilitate the absorption and desorption of water. When a solid surface comes into contact with an aqueous solution, it becomes charged due to the dissociation of surface charged groups, forming an electrical double layer (EDL) [17]. The counter ion cloud in the EDL moves with the flowing liquid, resulting in a streaming current. This phenomenon is mainly affected by surface charge, channel size, channel friction, etc. [16,33]. Therefore, the streaming potential can be increased by loading nano-carbon black particles on the surface of the wood to reduce channel diameter and increase surface electronegativity. The particle size of carbon black nanoparticles is about 10 nanometers, and the agglomerated particles when dispersed in the aqueous solution were also less than 200 nm (Figure S2). The nano-carbon black was uniformly loaded on the DW surface, and the pore surface changed from smooth to rough (Figure 2a,b and Figure S3). As a result, the micro-nanopores were formed (Figure S4). In this way, PEDOT:PSS would be uniformly loaded and the streaming potential would be improved as well. When coming into contact with water, the -OH, -COOH, and other functional groups on the surface of nano-carbon black particles dissociate hydrogen ions, forming a double layer (EDL) [34]. The loading of nano-carbon black particles was conducive to increase the surface electronegativity of fibers (Figure S5), which also promoted the streaming current. In addition, the mechanical properties of the loaded carbon black were also improved and enhanced its adaptability to practical applications (Figure S6).

PEDOT:PSS is considered to be a material with a unique position among the conductive polymers, which is widely used in conductive materials and mainly characterized by its high charge transfer capacity and environmental stability [35]. The PEDOT molecule itself is not easily soluble in water, and the -SO_3_H group in the PSS molecule serves as a disperser of PEDOT molecules to improve the solubility. Meanwhile, the -SO_3_H group can form hydrogen bonds and van der Waals forces with cellulose molecules and carbon black particles to improve the adhesion between PEDOT:PSS and carbon black wood [36]. PEDOT:PSS, a conductive polymer with excellent charge transfer ability, will provide an electron transfer platform for power generation, thus greatly reducing electron transfer resistance [37]. The six phosphate groups in PA molecules have a low dissociation constant (pKa = 1.5), which contributes to dissociating a large number of protons under hydration, increasing the number of mobile ions in the electric generator and further improving the output performance of MEG [38]. PA with chelating ability can chelate with PEDOT:PSS to replace part of the PSS chain, which enhances π–π stacking interaction and improves the conductivity of PEDOT (Figure 1b) [39,40]. Phytic acid can also generate van der Waals forces and hydrogen bonds with the active groups on the surface of carbon black nanoparticles, anchoring phytic acid molecules onto the surface of carbon black particles (Figure 2d). Meanwhile, PEDOT chelating with phytic acid will also firmly attach to the channel.

**Figure 3:**
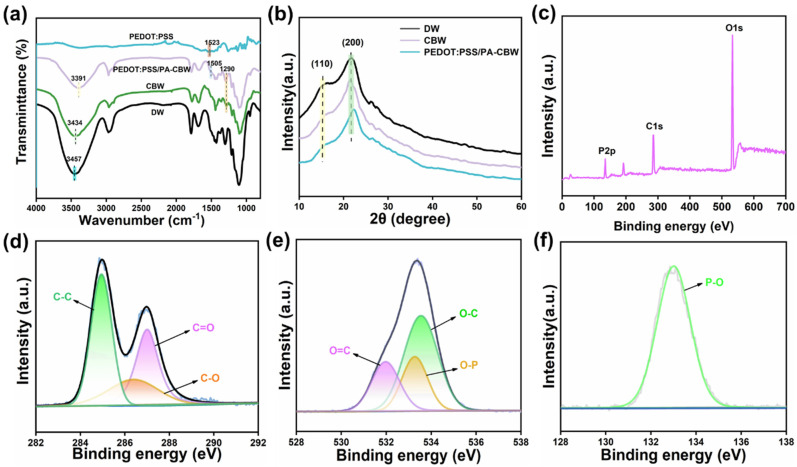
(**a**) FT-IR spectra of DW, CBW, PEDOT:PSS, and PEDOT:PSS/PA-CBW. (**b**) The XRD spectra. (**c**) XPS full spectrum. (**d**) Curve fitting of the C1 peak in the spectrum of PEDOT:PSS/PA-CBW. (**e**) Curve fitting of the O1 peak in the spectrum of PEDOT:PSS/PA-CBW. (**f**) Curve fitting of the P2p peak in the spectrum of PEDOT:PSS/PA-CBW.

The typical FT-IR spectra of DW, CBW, PEDOT:PSS, and PEDOT:PSS/PA-CBW are shown in Figure 3a. The peak at 3547 cm^−1^ in DW is caused by the symmetric stretching vibration of O-H in cellulose. Due to the hydrogen bonding between carbon black and cellulose, as well as between phytic acid and carbon black, both CBW and PEDOT:PSS/PA-CBW undergo a red shift, and the vibrational bands become wider. The peaks caused by the symmetric stretching vibration of O-H shift to 3434 cm^−1^ and 3391 cm^−1^, respectively. From Figure 2c,d, it can be seen that PEDOT:PSS is uniformly distributed on CBW. From the EDS spectrum in Figure 2e, the distribution of S and P elements can be seen, which also confirms the distribution of PEDOT:PSS and PA on CBW. According to Figure 3a, it can be seen that the C=C stretching vibration in the thiophene ring of PEDOT is located at 1523 cm^−1^. Due to the conjugation effect with the P=O bond in phytic acid, the delocalization of π electrons increases, resulting in a blue shift, and the stretching vibration peak also shifts to 1505 cm^−1^. As the addition of PA results in a higher peak intensity at this point than at CBW, the peak at 1290 cm^−1^ in PEDOT:PSS/PA-CBW comes from the P=O vibration absorption peak in phytic acid. The charged functional groups on the surface of carbon black (such as -C=OOH, -OH, -C=O) are the main contributors to the streaming current (Figure 3d–f), and the phosphate groups contained in phytic acid are also the main providers of the ion gradient diffusion current. The synergistic effect of the two greatly improves the output performance of the device. In addition, from Figure 3b, it can be seen that the addition of CB, PEDOT:PSS, and PA did not affect the crystal structure of cellulose.

### 3.1. Electricity Generation of MEG

**Figure 4:**
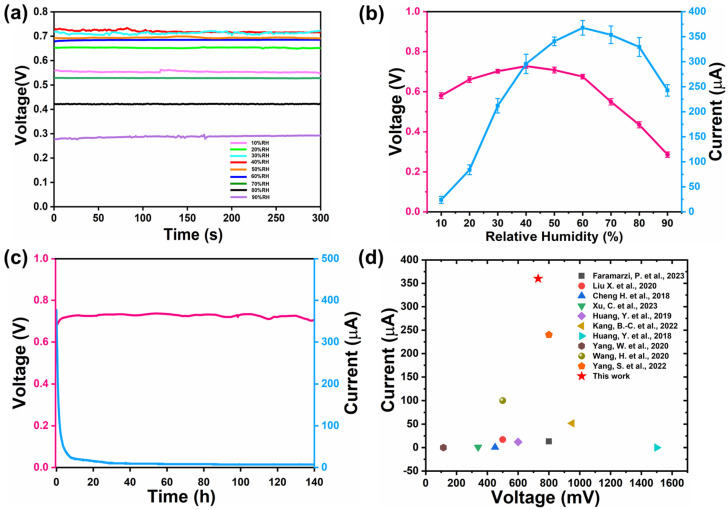
(**a**) Measured voltages of the PEDOT:PSS/PA-CBW MEG devices. (**b**) Measured voltages and current of the PEDOT:PSS/PA-CBW MEG devices. (**c**) A long-term recording of the voltage (red curve) and current (blue curve) from a generator for about 140 h. (**d**) Comparison of the voltage and current of a single component with various single generators reported previously for water- and moisture-enabled generation [13,19,38,41,42,43,44,45,46,47].

In order to explore the relationship between the output performance of the electric generator and humidity, we conducted output performance tests at a humidity range of 20–80% (Figure 4a,b). The electric generator had an area of 10 mm × 10 mm, with a thickness of 3 mm. At a relative humidity of 40%, the voltage reached a maximum of 0.728 V, while the current reached a maximum of 367 μA at a relative humidity of 50%. It was worth noting that the device also performed well in low ambient humidity. Even when the relative humidity of the environment dropped to 10%, there was still a voltage of 0.580 V and 27 μA. The current output reflects the strong all-weather working ability of the device. This is because LiCl is a highly hygroscopic salt that can quickly absorb water to form hydrated crystals when in contact with moist air and then form a salt solution. As H^+^ moves, Li^+^ also contributes to the output of MEG under the influence of a potential difference. The low-dissociation-constant characteristic of phytic acid causes it to ionize a large number of protons in contact with water. At low humidity, a large ion concentration difference could also be formed inside the electric generator, resulting in an increase in the potential difference and a larger voltage. As humidity increased, excessive water was absorbed. So, there was a decrease in the ion concentration difference and potential difference, resulting in a decrease in voltage. An increase in moisture would increase the current while reducing the internal resistance and transmission resistance of the generator. However, excessive moisture will balance the ion concentration on the high- and low-concentration sides, reduce the number of moving carriers, and lead to a decrease in the current. When the humidity is too low, the water absorbed by LiCl is insufficient, and the high ion concentration on the open electrode side cannot move to the closed electrode on the low-concentration side. Due to a lack of electrical pathways, the voltage is relatively low. The functional composite wood composed of CB, PEDOT:PSS, and PA generates good electrical signals and achieves a stable and continuous self-power supply. In Figure 4c, it can be clearly observed that MEG continuously and stably generated a voltage of 0.730 V, and a current exceeding 360 μA lasts for 140 h (approximately 5 days). Compared with other existing MEGs, the overall output performance of our equipment’s output voltage and current surpasses most electric generators in the past (Figure 4d) [13,19,38,41,42,43,44,45,46,47].

### 3.2. Power Generation Mechanism and Influencing Factors

**Figure 5:**
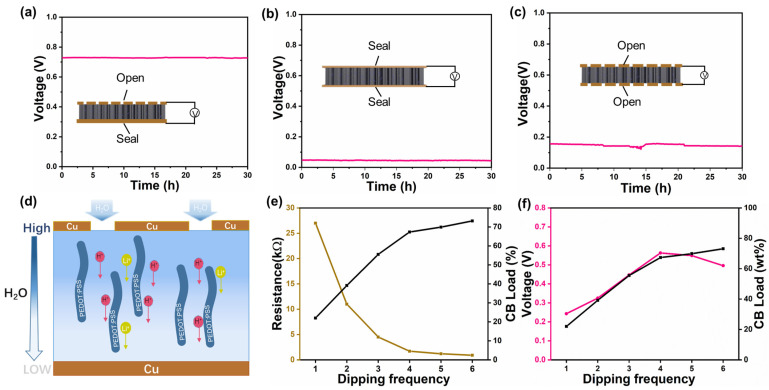
(**a**) The 30 h recording of voltage from a generator with one side sealed (temperature ≈ 25 °C, humidity ≈ 40% RH). (**b**) The 30 h recording of voltage from a generator in a completely sealed state (temperature ≈ 25 °C, humidity ≈ 40% RH). (**c**) The 30 h recording of voltage from a generator with both sides open (temperature ≈ 25 °C, humidity ≈ 40% RH). (**d**) Schematic diagram of moisture power generation of the device. (**e**) The load of different CB dipping frequencies and the voltage generated by CBW assembly into a generator. (**f**) CB loading and voltage of different impregnation times in CBW (temperature ≈ 25 °C, humidity ≈ 40% RH).

In order to verify that the output performance of MEG is closely related to the moisture and moisture gradient, the following experiments were conducted. As shown in Figure 5a, the MEG with an open electrode on one side generated a voltage of 0.730 V in an environment with a humidity of 40% RH. However, the voltage of MEG with open electrodes on one side and closed electrodes on both sides was reduced to several tens of millivolts (Figure 5b). It was obvious that the generation of a high voltage in MEG mainly relied on the humid air in the environment to provide a suitable environment for ion movement. We also conducted research on the impact of the humidity gradient on power generation. After replacing the MEG electrode with closed electrodes on both sides, the voltage decreased to 0.15 V, indicating that even with the presence of moisture, the high voltage would not be generated without a humidity gradient (Figure 5c). The magnitude of the humidity gradient had a significant impact on power generation.

When MEG was exposed to humid air, the hygroscopic LiCl would capture moisture in the air through the open electrode, while the closed electrode would not absorb moisture, thus establishing a humidity gradient from top to bottom. The hydrophilic oxygen-containing functional groups (-OH, -COOH) on the surface of carbon black would dissociate protons when in contact with water. Due to the effect of the electric double layer (EDL), protons moved with the liquid to form a streaming current [48,49,50]. At the same time, phytic acid dissociates a large amount of H^+^ in water. However, due to the difference in humidity between the open and closed sides, the ion concentration on the open electrode side was much higher than that on the closed electrode side, forming an ion concentration difference inside the MEG. Hydrogen ions moved in a directional direction under the mechanism of ion gradient diffusion, generating the current [51]. At the same time, with the help of the PEDOT:PSS electron transport layer, the overall output performance of the MEG was significantly improved (Figure 5d).

Carbon black nanoparticles also have a significant impact on the output performance of MEG. In Figure 5e, we immersed DW in a carbon black aqueous solution several times (the first, second, third, fourth, fifth, and sixth impregnations are, respectively, named CBW_1_, CBW_2_, CBW_3_, CBW_4_, CBW_5_, and CBW_6_) to increase the loading capacity of DW on carbon black particles. At the first impregnation with carbon black particles, it can be seen in Figure 5e that CBW_1_ has a high resistance. With the increase in the number of impregnations, the loading of carbon black particles on the surface of the fiber also increases, and the resistance of CBW_2_, CBW_3_, and CBW_4_ decreases rapidly. Until the fourth carbon black impregnation, the resistance of CBW_5_ and CBW_6_ did not change significantly, indicating that the carbon black particles can completely cover the fiber surface after four times of impregnation. When the carbon black particles completely cover the surface of DW, a continuous conductive path has been formed, and continuing to increase the loading of carbon black cannot lead to a significant decrease in resistance. When the carbon black particles completely covered the surface of the DW, a continuous path could not be formed and the decrease in resistance would be slow. As shown in Figure 5f, with the loading of carbon black particles, the MEG measurement voltage also continuously increased, reaching its maximum value during the fourth impregnation, and then slowly decreased. That is because carbon black particles did not fully cover the surface of the wood with fewer impregnations, making it impossible to form a continuous channel of carbon black particles and an effective conductive pathway. During the fourth impregnation, the surface of the wood was completely covered with carbon black particles, forming a good pathway for the occurrence of a high voltage. When the number of impregnations exceeded the fourth time, excessive loading of carbon black particles led to clogging of wood pores. Thus, the ion migration resistance increased, making it impossible to generate a high voltage. Similarly, it could be seen from the load capacity that the load capacity decreased relatively after the fourth impregnation. After the fourth impregnation, carbon black accumulated on the surface of the wood. It could be concluded that the CBW_4_ surface soaked four times with carbon black had good uniformity. Therefore, CBW_4_ samples were used for subsequent studies.

**Figure 6:**
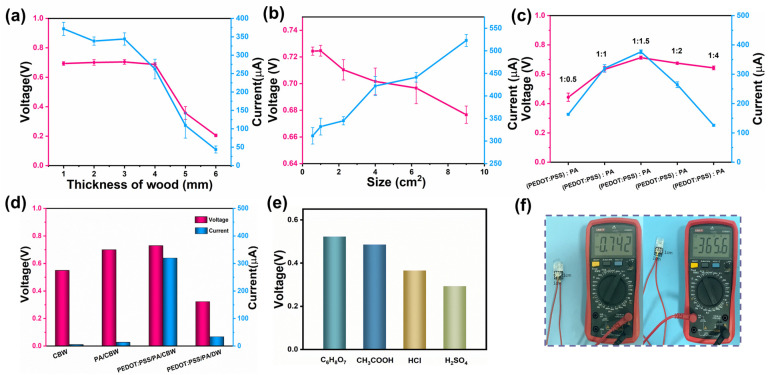
(**a**) Plot of Voc (red) and Isc (blue) versus device thickness at an ambient relative humidity of about 50% and a MEG size of 1.0 cm × 1.0 cm. (**b**) Plot of Voc (red) and Isc (blue) versus device size at an ambient relative humidity of about 50% and a MEG size of 1.0 cm × 1.0 cm. (**c**) The effect of PEDOT:PSS-to-PA ratio on output performance. (**d**) Output performance of generators with organic (citric acid, acetic acid) and inorganic (hydrochloric acid, sulfuric acid) acids added. (**e**) Output performance of CBW, PA/CBW, PEDOT:PSS/PA/CBW, PEDOT:PSS/PA/DW. (**f**) Physical diagram of generator size, voltage, and current.

This study investigates the impact of composite wood thickness on the output performance. The experiments were conducted with a consistent cross-sectional area of 1 cm^2^ (1 cm × 1 cm) for the composite wood samples. Results show that when the thickness exceeds 5 mm, the equipment fails to generate a sufficient voltage and current (Figure 6a). For devices with thicknesses ranging from 1 mm to 4 mm, the voltage remains around 0.720 V while the current slowly declines. This decline can be attributed to the remarkably elongated ion transport distance in thicker composite wood, which has a significant influence on the current magnitude. Consequently, a standardized thickness of 3 mm was adopted for subsequent investigations.

Furthermore, Figure 6b reveals an increasing trend in the current as the device size expands. This phenomenon can be explained by the abundance of microchannels within the device, where each channel can be regarded as an independent nanogenerator. Numerous tiny nanogenerators are integrated together to form the MEGs we have prepared. Consequently, enlarging the device size corresponds to an increase in the number of nanochannels, resulting in the integration of more tiny nanogenerators and generating a higher current. Conversely, the voltage does not exhibit significant variation with increased size. Therefore, improving the output performance merely requires scaling up the generator size.

The effect of the ratio of PEDOT:PSS and phytic acid addition on power generation was also studied. As shown in Figure 6c, as the proportion of phytic acid added increased, the voltage and current also increased. When the PEDOT:PSS and phytic acid ratio reached 1:1.5, the voltage and current were 0.723 V and 368 μA. When the proportion of PEDOT:PSS was relatively high, it would form gel due to the chelation of phytic acid, reducing its uniformity on the CBW surface [52]. At the same time, a small amount of phytic acid doping could not dissociate enough protons, so the output was not very good. When the proportion of phytic acid in the overall solution further increased, the voltage decreased more slowly and the current decreased significantly. That is because a decrease in the PEDOT:PSS ratio prevented the formation of a continuous ion transport layer in the conductive channel, resulting in a significant decrease in the current.

In order to verify the synergistic effect of carbon black, PEDOT:PSS, and PA leading to the high output of MEG, more studies were conducted (Figure 6d). After being loaded with carbon black, the voltage of CBW assembled into MEG is 0.550 V while the current is only 4 μA. After doping PA in CBW, the voltage reached 0.690 V, and the current only increased to 15 μA. The large number of protons dissociated from PA increased the potential difference of MEG. However, due to the lack of a good electron transport layer, the current had not been significantly increased. We obtained a MEG device by adding PEDOT:PSS and PA in a ratio of 1:1.5 to CBW_4_. The voltage measured after assembly reached 0.730 V, and the current increased by 360 μA. It can be seen that PEDOT:PSS was of indispensable importance for improving the generation current. In addition, we also filled PEDOT:PSS and PA in DW at a ratio of 1:1.5, and assembled them into MEG. The measured voltage and current were only 0.330 V and 45 μA, respectively. It shows that the carbon black particles loaded on the surface of DW play a role in reducing the overall internal resistance and ion transport resistance. To verify the irreplaceability of phytic acid, we replaced the added phytic acid in MEG with organic acids (citric acid, acetic acid) and inorganic acids (hydrochloric acid, sulfuric acid) of the same concentration for output performance testing. From Figure 6e, it can be seen that after replacing PA with other acids, the high voltage output cannot be achieved. The main reason was that there were 12 dissociable protons in the phytic acid molecule, which formed a large ion concentration difference. The protons produced through the dissociation of other acids failed to achieve this large ion concentration difference. We utilized the power generation mechanism of MEG equipment: the streaming current and ion diffusion mechanism. Through the synergistic effect of CB, PEDOT:PSS, and PA, we successfully prepared MEG with excellent performance by increasing the number of movable ions and reducing channel transmission resistance as much as possible for MEG (Figure 6f).

### 3.3. Practical Application

**Figure 7:**
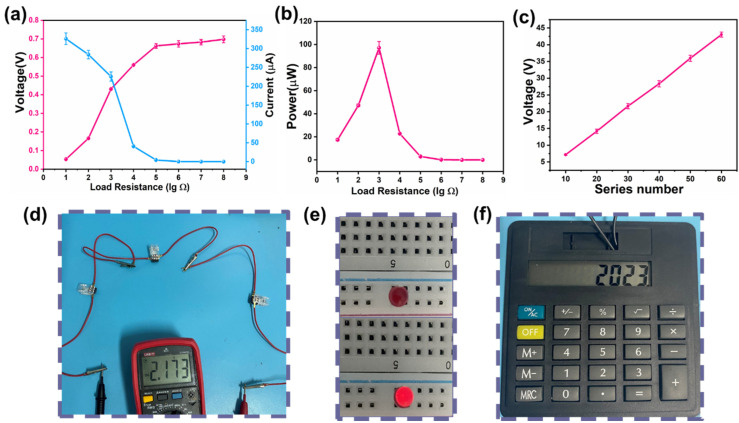
(**a**) Voltage and current measured at different load resistances. (**b**) Output power of HEG measured at different load resistances. (**c**) Linear relationship between number of MEGs and voltage. (**d**) Integration of 3 units of MEG measured using commercial multimeter in voltage mode; (**e**) led bulbs are lit by 3 units of integrated MEGs. (**f**) The integrated 3-unit MEGs power the calculator for proper operation.

In terms of practical application, output power is an important indicator for evaluating MEG. Figure 7a shows that as the load resistance increased from 10 Ω to 10^8^ Ω, the voltage gradually increased and the current gradually decreased. According to the effective power calculation formula, P = UI [53], we can see that when the load resistance was 1.8 KΩ, the maximum power reached 97 μW (Figure 7b). According to different application requirements, multiple MEG units in series or parallel can drive various electronic devices. For example, three MEGs connected in series provided a voltage of 2.1 V (Figure 7d), which can light up a single LED without any rectification equipment or capacitors, and was also sufficient to drive a commercial calculator (Figure 6e,f, and Movies S1 and S2). Expandable integration is crucial for MEGs, as it can expand the device’s output through simple series integration. One MEG unit generated a voltage of 0.72 V, and 10 MEG units connected in series obtained a voltage of 7.2 V (Figure S7). By integrating 60 units of MEG in series, the voltage output reached 43 V (Figure 7c). The voltage output exhibits a good linear relationship with the number of devices, allowing for easy scaling up or down, demonstrating a good linear relationship.

## 4. Conclusions

In summary, we modified the surface of wood pores and constructed CB-loaded functional wood with a micro-, nano-scale pore structure, achieving effective water transfer. A moisture absorbing and continuous conductive network in wood fibers was prepared by impregnating functional wood with PEDOT:PSS/PA, allowing charge carriers to move rapidly in the PEDOT:PSS conductive layer, and thus providing higher current output for MEG. A single device had an open circuit voltage of 0.730 V and a current exceeding 360 μA without external auxiliary conditions. It can maintain a continuous and stable output of over 72 h under daily humidity, surpassing the output performance of most humidity generators. The MEG only requires simple series or parallel integration to provide higher output performance. This study provides guidance for improving the output performance of generators made of biomass materials and provides a new approach for the application of wood in self-generating equipment.

## Data Availability

The data presented in this study are available on request from the corresponding author.

## Supplementary Materials

The following supporting information can be downloaded at: https://www.mdpi.com/article/10.3390/polym16020260/s1, Figure S1: The content of cellulose, hemicellulose, and lignin in NW and DW; Figure S2: Particle size distribution of CB; Figure S3: SEM images of DW; Figure S4: The aperture size of CBW; Figure S5: Zeta potential of CB surface; Figure S6: Compression properties of NW, DW, and CBW; Figure S7: Photograph shows 10 units of MEG in series, which can boost the output voltage to 7.2 V. Movies S1 and S2.
