# Supplementary material for: Modified Wood Fibers Spontaneously Harvest Electricity from Moisture
Source: Polymers (Basel). 2024 Jan 17;16(2):260. doi: 10.3390/polym16020260 (PMC10818770; doi:10.3390/polym16020260)
Supplement: Supplementary file 1 [file polymers-16-00260-s001.zip › supplementary.docx]

Supplementary Material

Modified wood fibers spontaneously harvest electricity from moisture

Tao Zhang, Xuewen Han, Yukang Peng, Han Yu, Junwen Pu*

Beijing Key Laboratory of Lignocellulosic Chemistry, Beijing Forestry University, Beijing 100083, China

* Correspondence: jwpu@bjfu.edu.cn; Tel.: +86-0592-6167377; +86-10-62336163


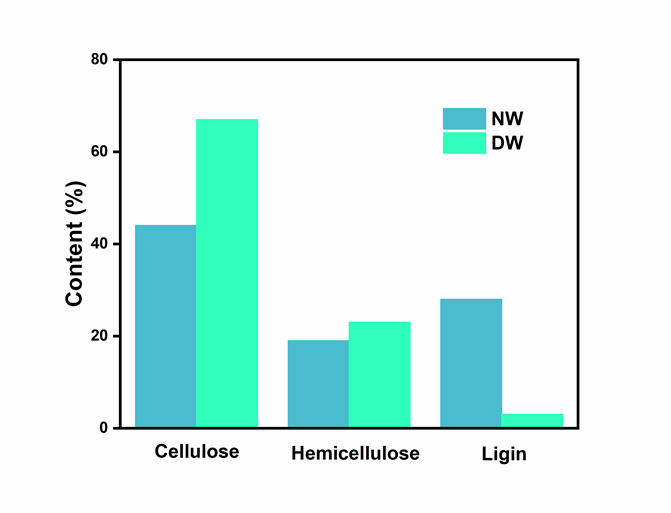


**Figure S1.**The content of cellulose, hemicellulose and lignin in NW and DW.


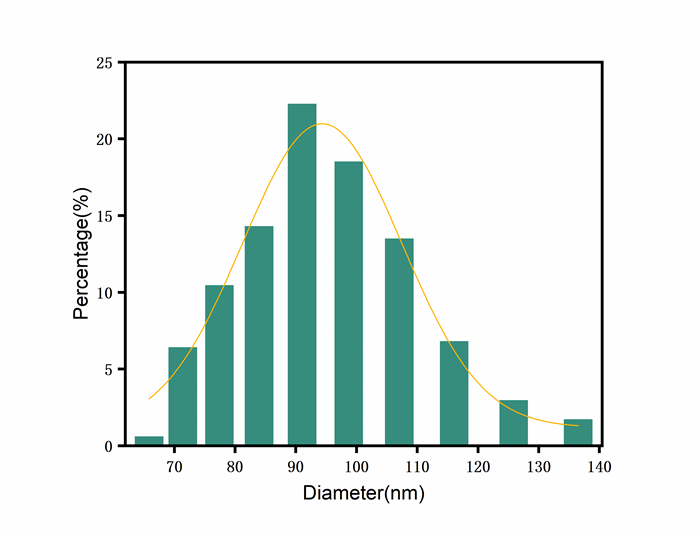


**Figure S2.** Particle size distribution of CB.


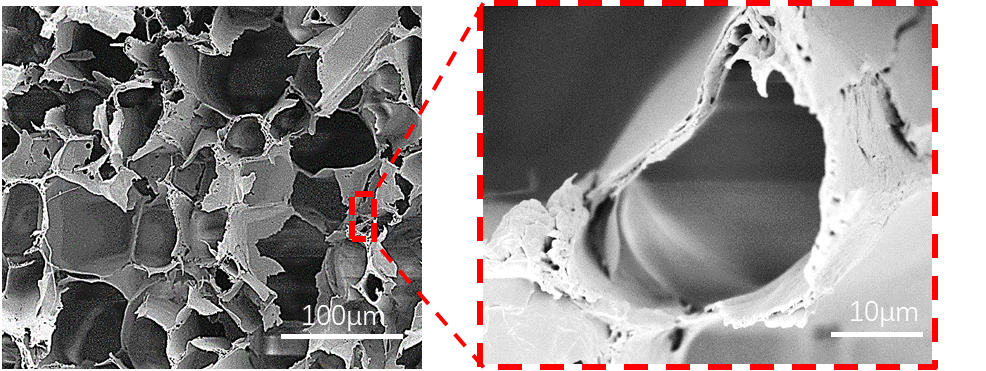


**Figure S3.** SEM images of DW.


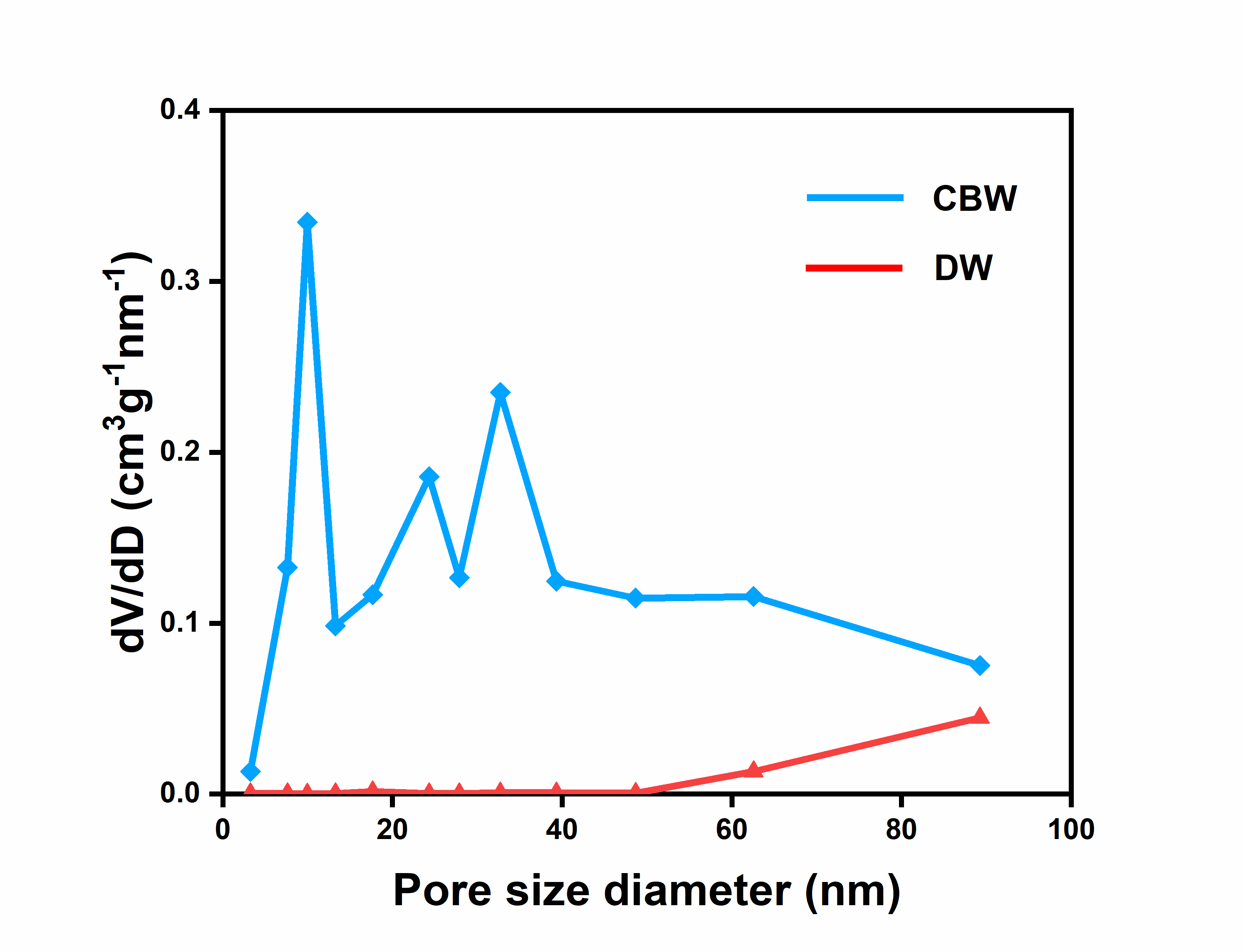


**Figure S4.** The aperture size of CBW.


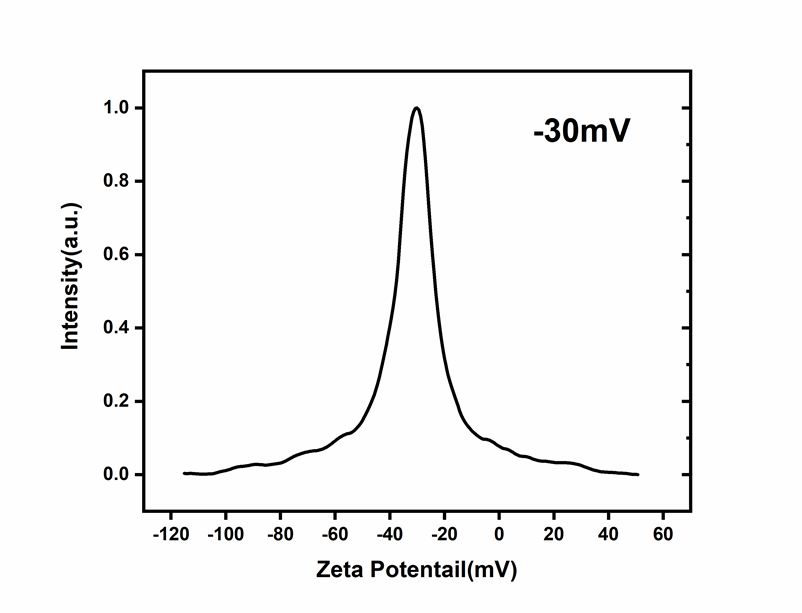


**Figure S5.** Zeta potential of CB surface.


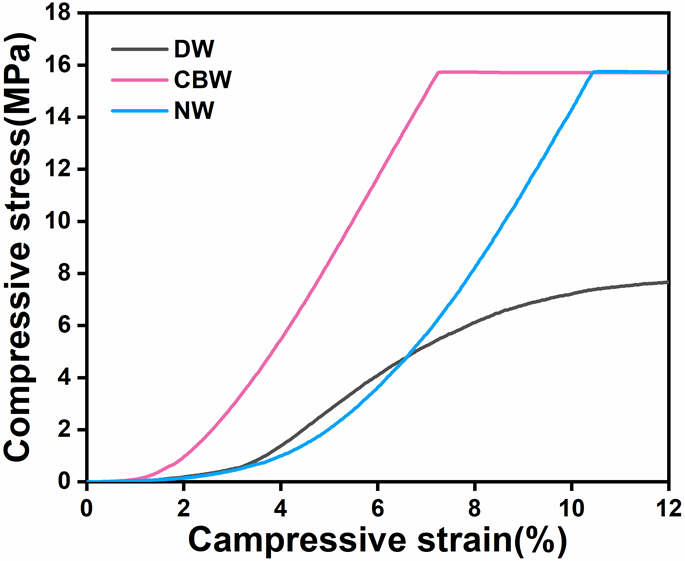


**Figure S6.** Compression properties of NW, DW and CBW.


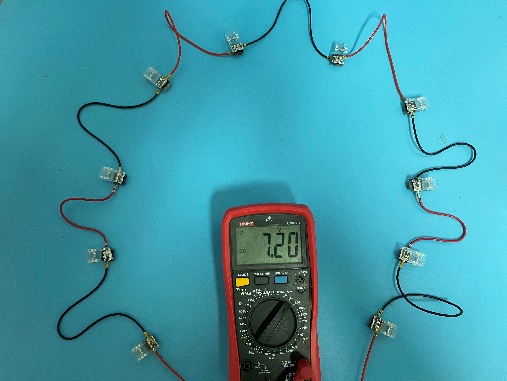


**Figure S7.** Photograph shows 10 units of MEG in series, which can boost the output voltage to 7.2V.
